# Magnetic UiO-66 functionalized with 4,4′-diamino-2,2′-stilbenedisulfonic as a highly recoverable acid catalyst for the synthesis of 4*H*-chromenes in green solvent

**DOI:** 10.1038/s41598-022-09337-z

**Published:** 2022-04-01

**Authors:** Mohammad Reza Khodabakhshi, Mohammad Hadi Baghersad

**Affiliations:** Applied Biotechnology Research Center, Baqiyatallah University of Medical Sciences, Tehran, Iran

**Keywords:** Materials science, Nanoscience and technology, Chemistry, Organic chemistry

## Abstract

According to 4*H*-chromenes importance, we synthesized a novel magnetic UiO-66 functionalized with 4,4′-diamino-2,2′-stilbenedisulfonic as an efficient and reusable solid acid catalyst for synthesizing 4*H*-chromene skeletons via a one-pot three components reaction in a green solvent. The structure of the synthesized catalyst was confirmed by various techniques including FT-IR, XRD, BET, TGA, TEM, EDX, and SEM, and also the product yields were obtained in 83–96% of yields for all the reactions and under mild conditions. The reported procedure presents an environmentally friendly approach for synthesizing a significant number of 4*H*-chromene derivatives. Correspondingly, MOF-based catalyst makes it easy to separate from reaction media and reuse in the next runs.

## Introduction

**Figure 1:**
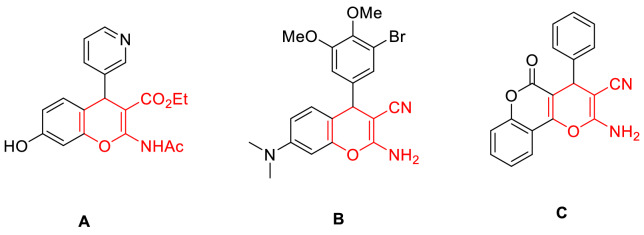
Structures of some biological active 4*H*-chromenes.

4*H*-chromenes can be found in various natural compounds, such as biologically and therapeutically active drugs (anticonvulsants, antimicrobial, and anticancer agents) (Fig. 1)^1^. Researchers have developed several methods for synthesizing 4*H*-chromene derivatives, including using one-pot synthesis methods, recyclable catalysts, green methodologies (reactions in aqueous media), catalyst utilization, and byproduct eliminations^2–6^.

Numerous catalytic systems have been developed till today for the synthesis of 4*H*-chromene derivatives, consist of Fe(HSO4)_3_^7^, nickel nanoparticles^8^, ZrO_2_ nanoparticles^9^, Zn_4_O(H_2_N-TA)_3_^10^, ZnS nanoparticles^11^, nano-sized MgO^12^, CoFe_2_O_4_^13^, CuO-CeO_2_^14^, polymer-supported palladacycles^15^, [2-aemim][PF_6_]^16^ IL-HSO4@SBA-15^17^, SB-DBU.Cl^18^, potassiumphthalimide-*N*-oxyl^19^, tungstic acid functionalized mesoporous SBA-15^20^, heteropolyacid^21^, Mg/Al hydrotalcite^22^, PEI@Si–MNP^23^, PEG-SO_3_H^24^, alumina^25^, nano-sized zeolite clinoptilolite^26^, Nickel Nanoparticles^8^, (CTA)_3_[SiW_12_]-Li^+^-MMT^27^, PMO-ICS^28^, poly(N,N′-dibromo-Nethyl-benzene-1,3-disulfonamide (PBBS)^29^, KSF^30^, combined NaOAc/KF^31^, MeSO_3_H^32^, TiCl_4_^33,34^, MA liquid-phase^35^, Bovine Serum Albumin^36^, and Cysteic acid grafted to magnetic graphene oxide^37^.

However, some of the catalysts for the synthesis of 4*H*-chromenes suffer from disadvantages such as making pollution, having high cost, having difficulty in removing catalysts, and demanding harsh reaction conditions. According to the importance and the broad application of *4H*-chromenes, there is still a great demand for a more feasible, simple, green, and efficient way to synthesize these compounds. For these reasons, we try to design heterogeneous magnetic catalysts to synthesize 4*H*-chromenes with MOFs substrate.

Porous coordination polymers (PCPs), also known as metal–organic frameworks (MOFs), have attracted many scientists' attention during recent years^38–43^. The structure of MOFs can be revised and planned in many ways, considering three main factors: clusters of metal ions, inorganic metal ions, and organic linkers^44–47^. Researchers have designed, synthesized, and commercialized novel MOFs and studied their applications for the last two decades^48–54^. Since MOFs have significant advantages, such as adjustable pore size and functionalities, appropriate capacity for adsorption, considerable specific surface area, and low density, controllable pore functionalities, they can be widely used for adsorption and removal of dyes^55–57^.

Nevertheless, some MOFs showed vital negative points, like poor chemical stability. These negative points lead to various limitations in using MOFs’ possibilities. To overcome the negative points of MOFs’, various functional materials were combined to enhance their ability^58–61^. The synthesis of hybrid nanomaterials based on magnetic nanoparticles and MOFs^62^ are of these combinations. These kinds of combinations make it possible to use the advantages of both components, such as high chemical stability and simple separation process, for different applications, especially enhancements in the kinetics of adsorption^63–66^.

In detail, magnetic nanomaterials can act as effective adsorbents due to their ease of removing contaminants from wastewater by an applied magnetic field. Also, bio-sorbents have a synergistic effect with their efficient adsorption capacity to remove contaminants, to participate in waste minimization^67,68^, and to aid alleviate ecological complications^69^. For these reasons, resulted MOFs from these combinations possess interesting characteristics that could work adequately in CO_2_ carbon capture. But the main disadvantage of using MOFs as adsorbents in CO_2_ carbon capture is the energy-intensive nature connected with the desorption progression (sunlight, as a powerful external stimulus, can enable the desorption progression with much lesser energy demand over MOF materials). In these occasions, computational screening modeling approaches are influential tools to find optimum performing materials. With the aid of computational modeling, synthesized Mg-IRMOF-74-III showed a CO_2_ adsorption capacity of 89.6 cm^3^ g^−1^, which is the highest CO_2_ adsorption value within photo-responsive MOFs compared to the reported literatures^70^.

As mentioned above, due to magnetic nanoparticles' efficiency, the disadvantage of MOFs could vanish by various methods, like combining the MOFs and magnetic particles. Magnetic hybrid MOFs presented sizeable specific surface areas for their easy separation method. Several methods have been studied till today for the synthesis of magnetic MOFs. These methods include combining the MOFs with Fe_3_O_4_ by a simple method, coating MOFs onto Fe_3_O_4_ using layer-by-layer strategy, embedding Fe_3_O_4_ into MOFs, and encapsulating Fe_3_O_4_ into MOFs^71–79^. Among all the methods, synthesizing magnetic MOFs using a step-by-step method is one of the most reliable ways. The adjustability of the thickness of the outer shell MOFs is one of the main advantages of this method. Some adjustments are required to develop the compatibility of shell and core and gain the best results^80^.

Stilbenes are a class of secondary metabolites containing a trans/cis-ethene double bond and a phenyl on each of the double-bond carbon atoms. The majority of stilbenes are thermally-chemically stable. Additionally, they show fluorescence properties and absorption abilities^81,82^. They play in many required fields such as biomedical^71^, biophysical^67^, and photochemical research^63^. Due to their applications in a wide range of branches, stilbenes can be used in multidisciplinary fields and syndicates biology, medicine, physics, and chemistry together^83–86^. They are promising agents for use as a functional group for catalytic uses. As the other derivatives of aromatic sulfonic acids, stilbene sulfonic acids are also used to prepare optical brighteners and synthetic dyes^87,88^.

**Figure 2:**
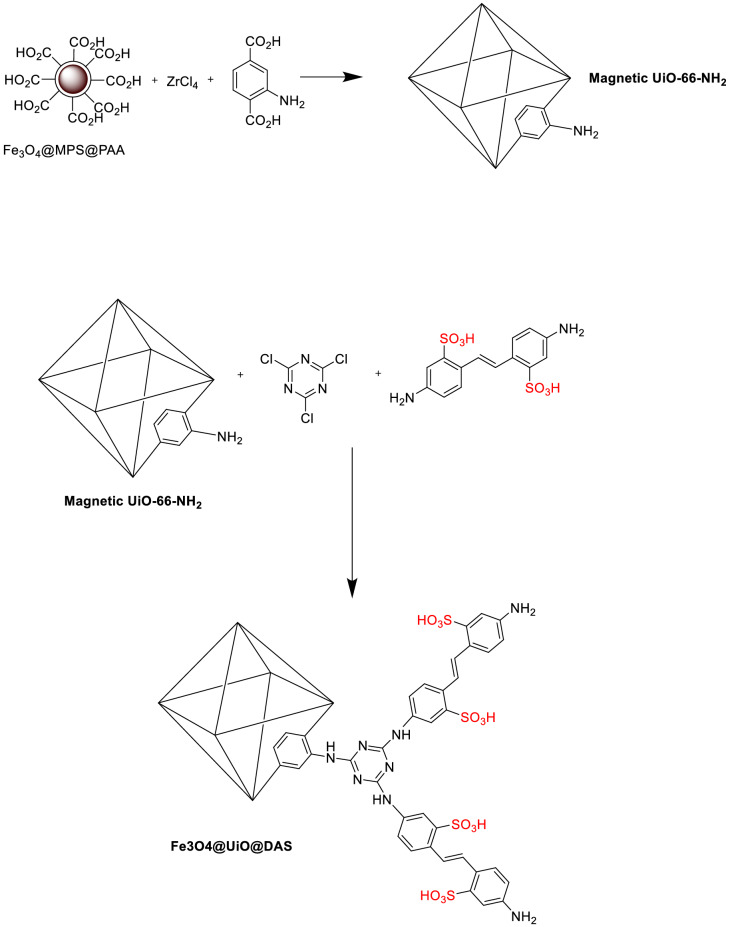
The Synthesis procedure of the Fe_3_O_4_@UiO@DAS.

**Figure 3:**
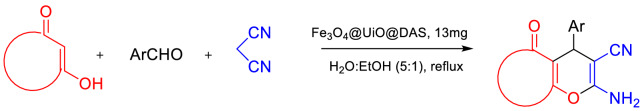
Synthesis of 4*H*-Chromene derivatives catalyzed by Fe_3_O_4_@UiO@DAS.

In this project, to investigate the applications of recoverable solid acid catalysts for the synthesis of 4*H*-chromenes, Zr clusters with 4,4′-Diamino-2,2′-stilbenedisulfonic were used to design modified magnetic MOF. Zr-cluster-based MOFs, like UiO-66 and UiO-67, have fascinating acid, thermal, and aqueous stabilities^89–94^. Due to their wide range of applications, we have synthesized UiO-66 (Figs. 2, 3) to study its application as a catalyst^95–97^ in the synthesis of 4*H*-chromene skeletons via a one-pot three components reaction. The product yields were obtained in 83–96% of yields for all reactions. Studies showed that acidic reagent plays the main role in the catalytic cycle in these reactions.

## Results and discussion

The Fe_3_O_4_@UiO@DAS catalyst was synthesized using a few steps presented in Fig. 2. Details of the preparation method are described in the experimental section.

**Figure 4:**
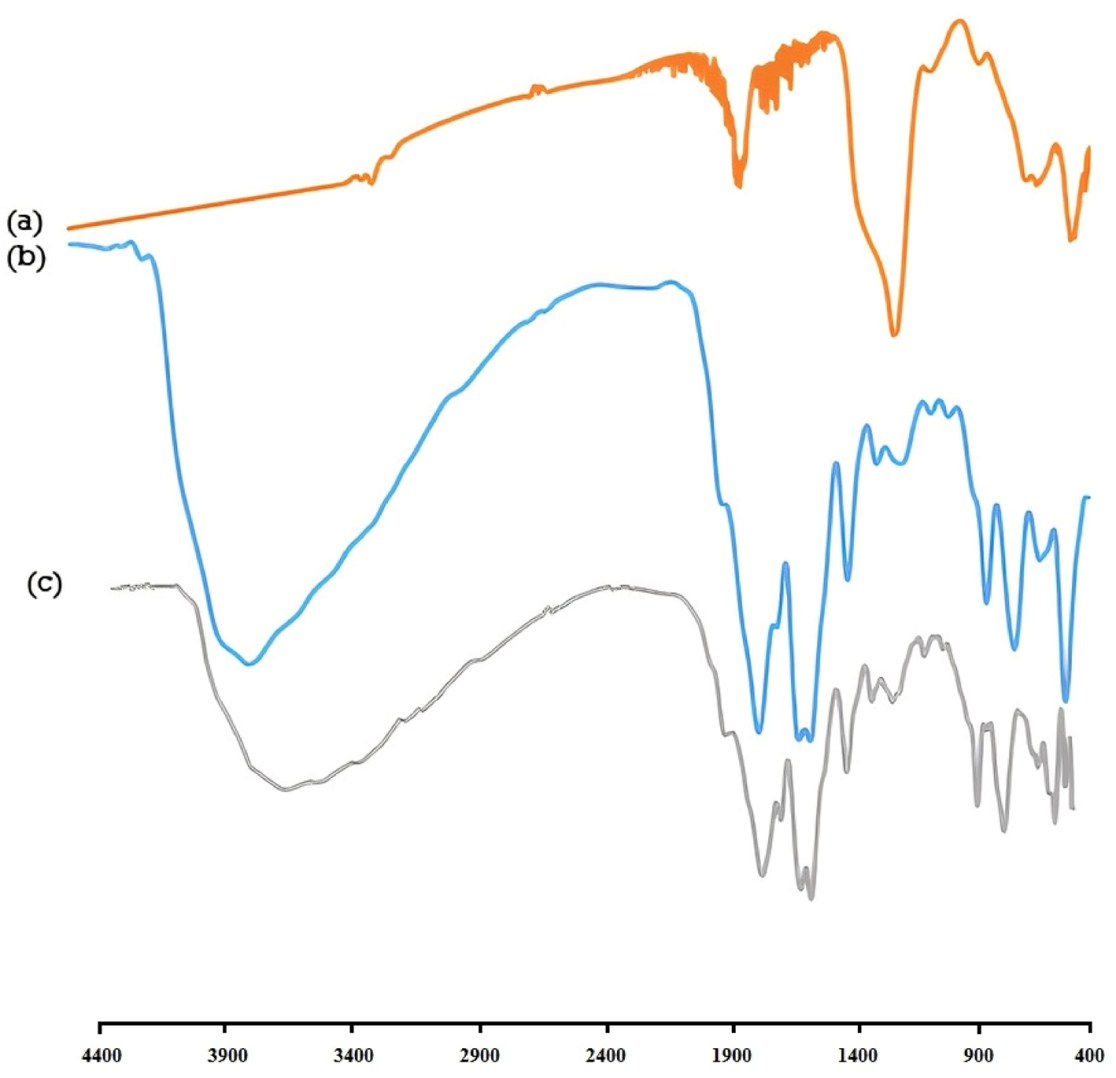
(**a**–**c**) The FTIR spectrums.

The FTIR spectrum of Fe_3_O_4_ (Fig. 4a), Fe_3_O_4_@UiO-66 can be seen in Fig. 4b. In this figure, the Fe–O band is appeared at 630 cm^−1^ (due to the presence of Fe_3_O_4_), two peaks at around 1088 cm^−1^ are due to the presence of S–O (stretching vibrations), the peak at 1634 and 1709 cm^−1^ is attributed to C=C and C=O bands, respectively, the C–H bands can be seen at 2931 cm^−1^, and the strong broad bands at 3435 cm^−1^ can be assigned to stretching of O–H (for Fe_3_O_4_).

In the spectra of final product Fe_3_O_4_@UiO@DAS in Fig. 4c, in addition of mentioned peaks for Fe_3_O_4_@UiO-66, C–N peaks at 1502 and 1573 cm^−1^ can be seen. Also, aromatic peaks are appeared below 1000 cm^−1^.

**Figure 5:**
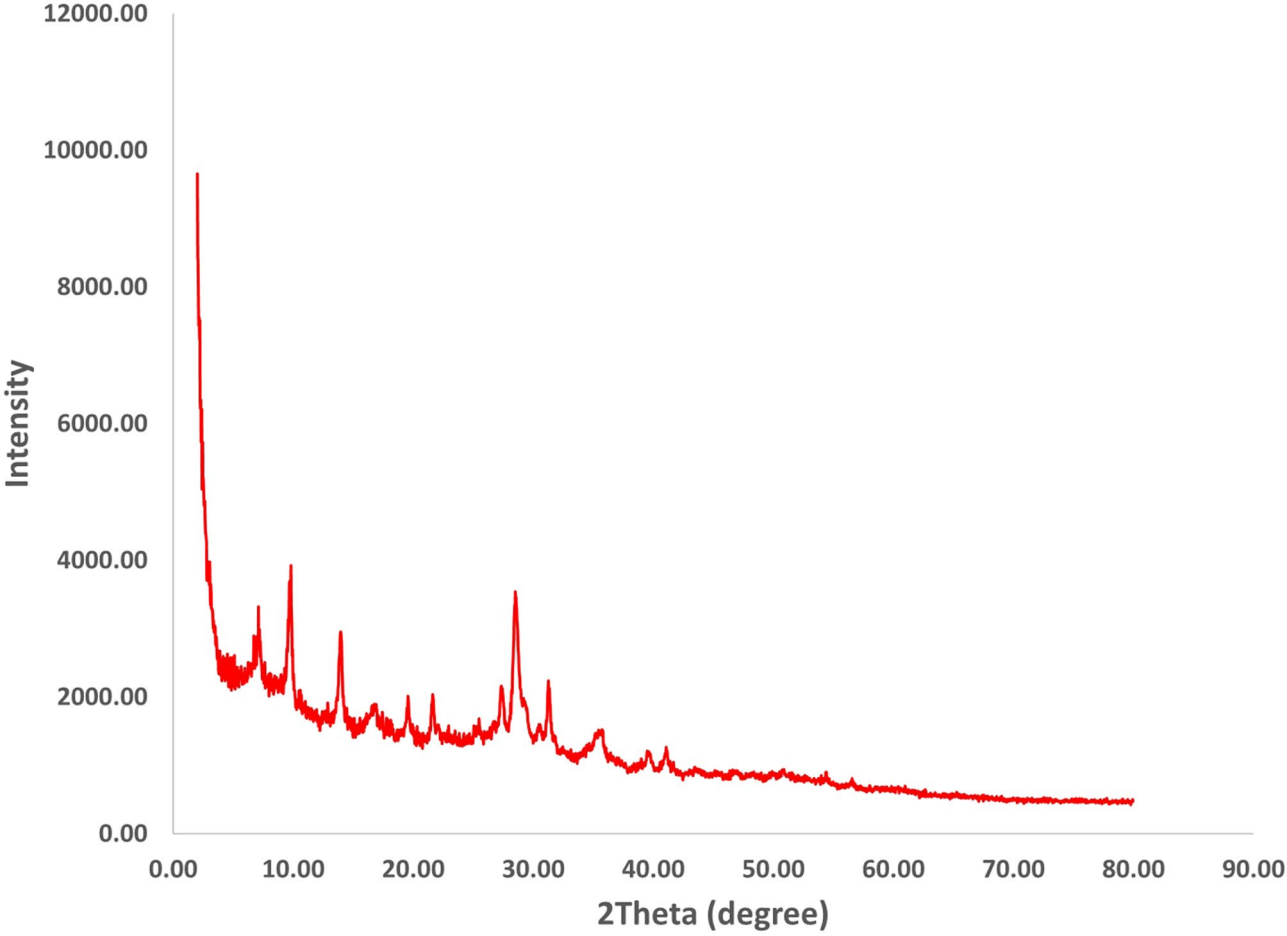
XRD pattern of Fe_3_O_4_@UiO@DAS.

In the XRD analysis of Fe_3_O_4_@UiO@DAS (Fig. 5), observed diffraction peaks are similar to UiO-66 pattern which was reported before^98–100^. In this pattern, not any apparent variations in the characteristic diffraction pattern of Fe_3_O_4_@UiO-66 were observed. This shows that after growing on the surface of functionalized Fe_3_O_4_ nanoparticles, the crystalline structure of the MOF was remained unchanged^99,100^.

**Figure 6:**
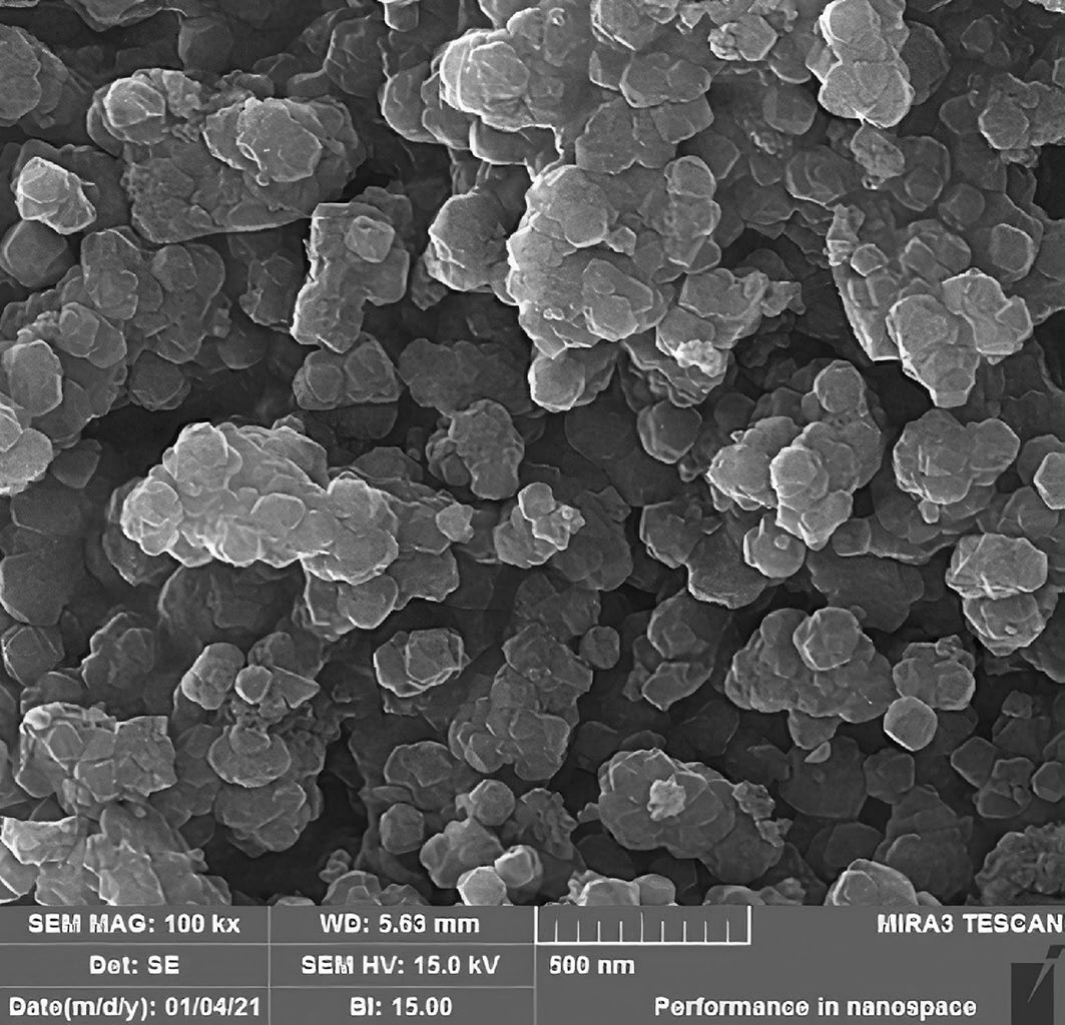
SEM analysis of Fe_3_O_4_@UiO@DAS.

**Figure 7:**
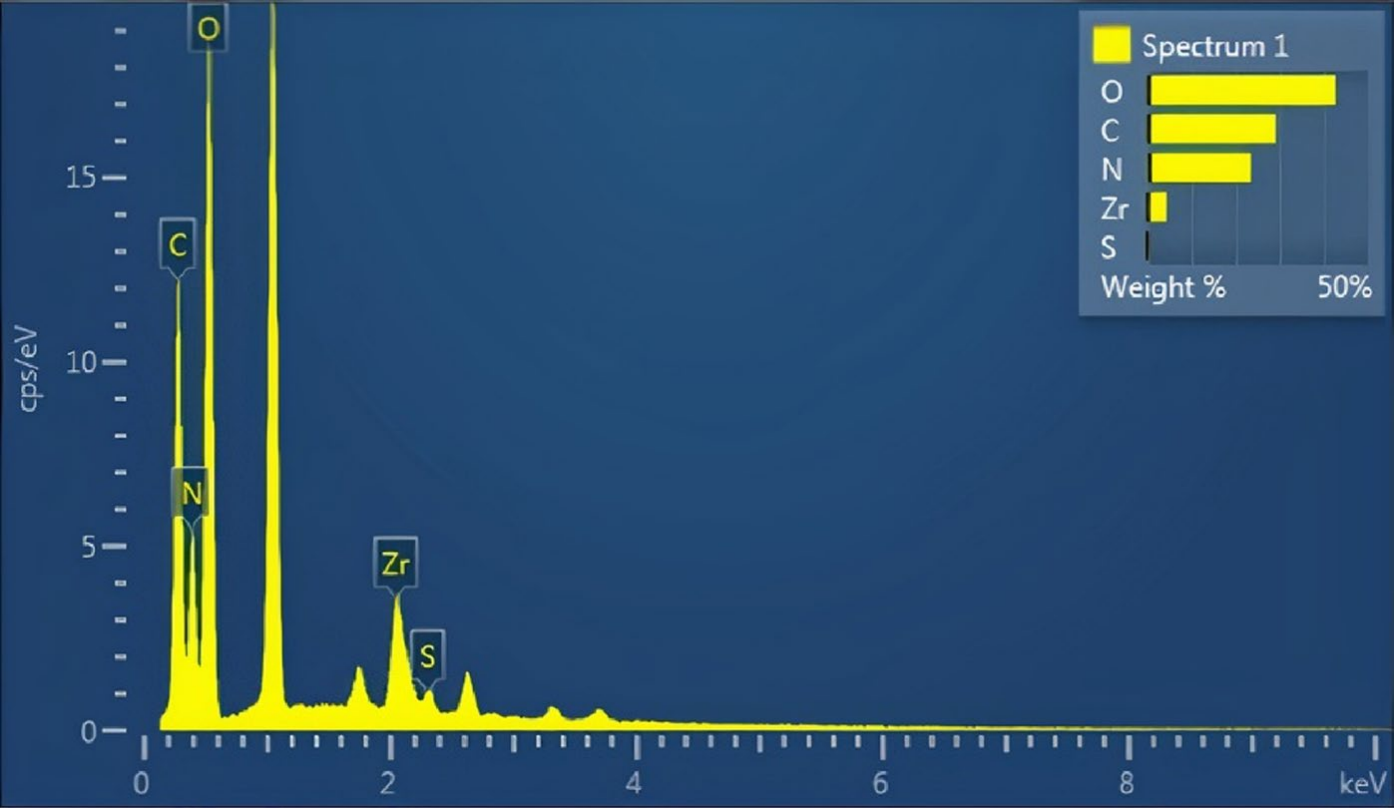
EDX analysis of Fe_3_O_4_@UiO@DAS.

**Figure 8:**
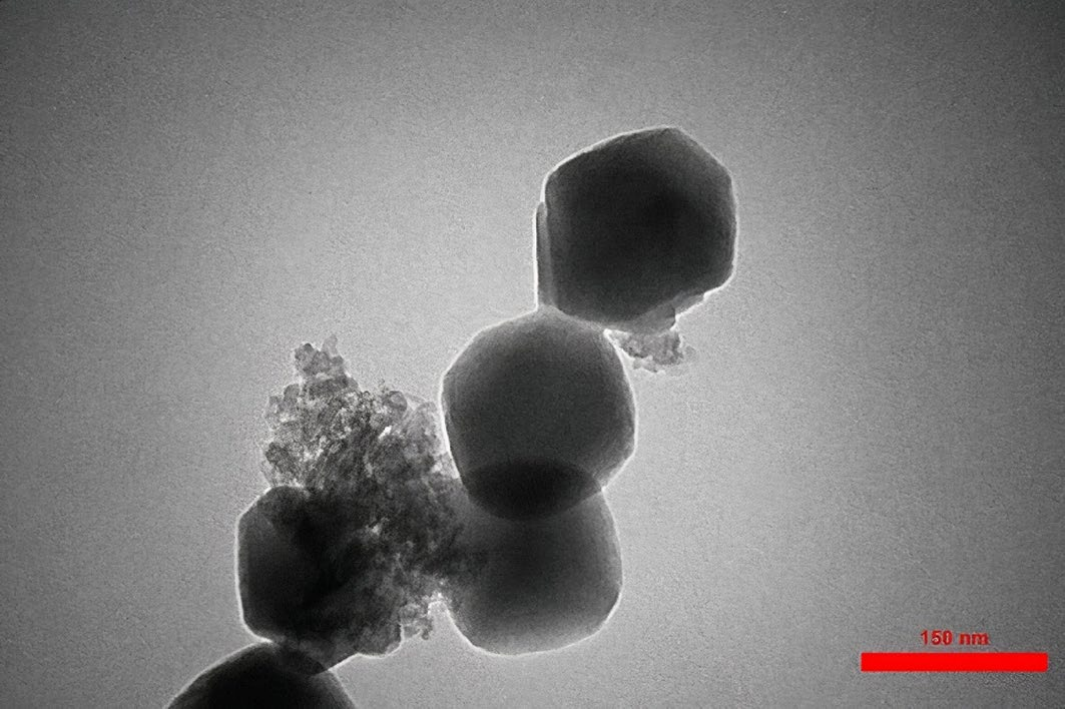
TEM analysis of Fe_3_O_4_@UiO@DAS.

To study the morphology, size, and also structure of Fe_3_O_4_@UiO@DAS, SEM and TEM analyses were used (Figs. 6, 7, 8). The SEM images can show the particle size (by randomly selected particles and studying the size distribution of them) and also illustrated that the particles have a cubic structure. TEM images of the prepared MOF show good agreement with other literatures and can confirm the Fe_3_O_4_ core of the obtained catalyst. Additionally, using the EDX pattern of the synthesized catalyst, the main elements in its structure (Fe, O, C, N, S) could be understood. These analyses proved the successful synthesis of our catalyst. From EDX we can understand the different amount of carbon in our final catalyst (around 30 weights % in Fe_3_O_4_@UiO@DAS) from our initial samples.

**Figure 9:**
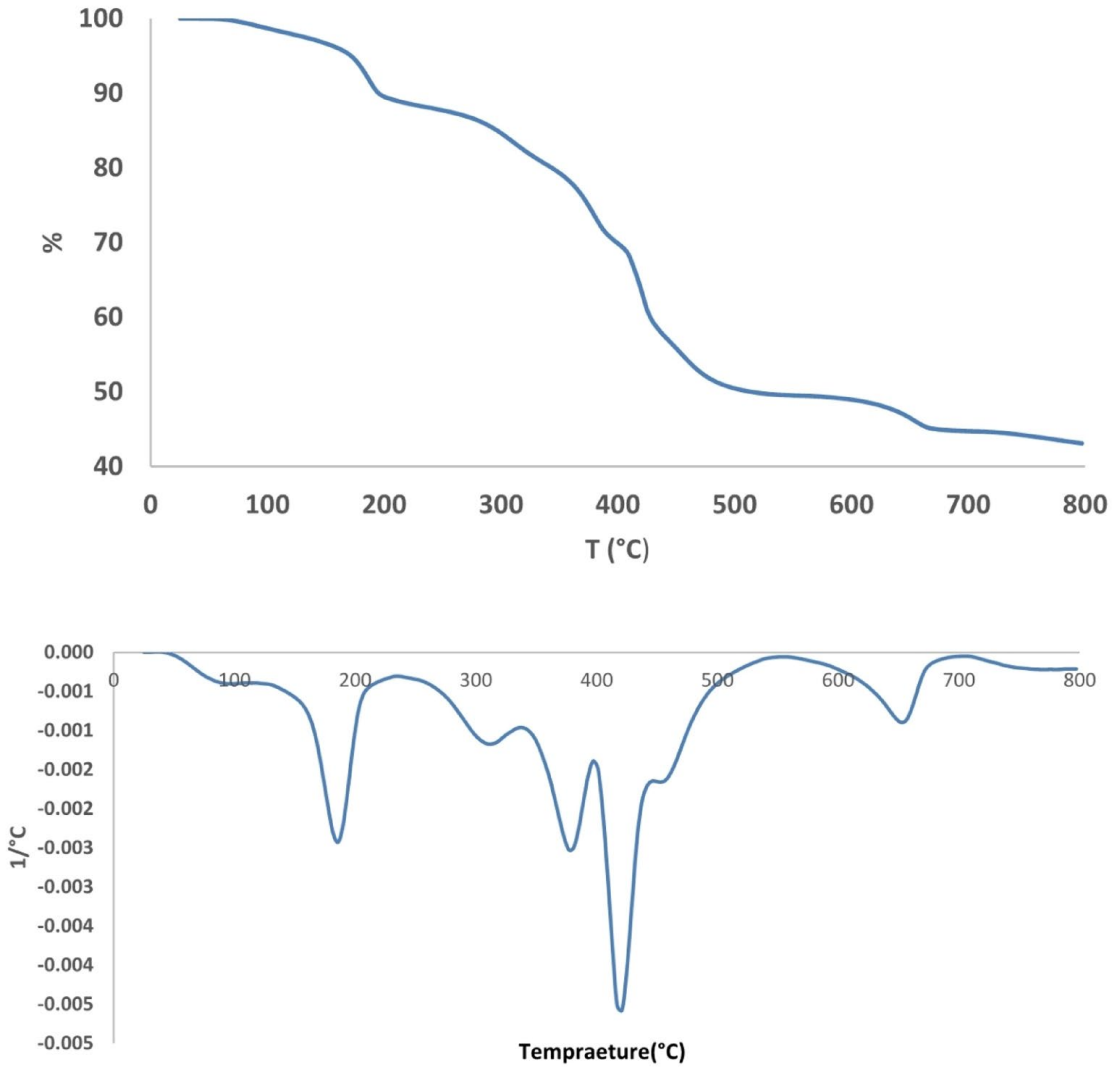
TGA-DTG analysis.

TGA analysis shows the thermal stability of the synthesized catalyst (Fig. 9). The first decomposition was placed between 100 and 200 °C, due to the trapped water. The second stage, between 260 and 330 °C, is attributed to the decomposition of 4,4′-diamino-2,2′-stilbenedisulfonic acid. Next, the other weight losses that occurred at around 350–390 and 400–420 °C, are because of the removal of hydroxyl, sulfonic acid, and carboxylic acid groups. In higher degrees, owing to the presence of Fe_3_O_4_, the line becomes stable with no considerable changes.

The N_2_ adsorption–desorption data have been summarized in Table 1. The BET specific surface areas of magnetic Fe_3_O_4_@UiO-66 and Fe_3_O_4_@UiO@DAS are 828 and 725 m^2^ g^−1^, respectively.

**Table 1:** N_2_ adsorption–desorption data.

Sample	Total pore volume (cm^3^ g^−1^)	BET surface area (m^2^ g^−1^)	Pore diameter (nm)
Fe_3_O_4_@UiO-66	0.41	828	5.1
Fe_3_O_4_@UiO@DAS	0.38	725	4.8

The catalytic ability of the synthesized catalyst was studied through the synthesis of 4*H*-chromene derivatives. To find the optimum reaction condition, the reaction of benzaldehyde (1.0 mmol) (**1**), malononitrile (1.1 mol) (**2**), and 4-hydroxy-6-methyl-2*H*-pyran-2-one (1.0 mmol) (**3**) were studied in various conditions (Table 2). First, different solvents include H_2_O, EtOH, EtOH (1):H_2_O (5), and THF in the absence of the catalyst. The best yield (29%) was belonged to EtOH (1):H_2_O (5) combination (29% yield, 6 h) (Table 2, entries 1–4). Based on the literature, the result shows that a catalyst is necessary to improve the desired reaction rate and yield.

**Table 2:** Optimization of the reaction condition for the synthesis of 2-amino-3-cyano-4*H*-chromene catalyzed by Fe_3_O_4_@UiO@DAS.


Entry	Cat. (mg)	Temp	Solv	Time (h)	Yield^a^ (%)
**1**	–	Reflux	H_2_O	6	18
**2**	–	Reflux	EtOH	6	21
**3**	–	Reflux	H_2_O:EtOH (5:1)	6	29
**4**	–	Reflux	THF	6	Trace
**5**	4,4′-Diamino-2,2′-stilbenedisulfonic acid **3** mg	Reflux	H_2_O:EtOH (5:1)	6	73
**6**	MNP@MPS **3** mg	Reflux	H_2_O:EtOH (5:1)	6	32
**7**	MNP@MPS@PAA **3** mg	Reflux	H_2_O:EtOH (5:1)	6	45
**8**	Fe_3_O_4_@UiO-66-NH_2_ **3** mg	Reflux	H_2_O:EtOH (5:1)	6	32
**9**	Fe_3_O_4_@UiO@DAS **5** mg	Ambient	Solvent-free	6	Trace
**10**	Fe_3_O_4_@UiO@DAS **5** mg	80	Solvent-free	6	57
**11**	Fe_3_O_4_@UiO@DAS **5** mg	Reflux	H_2_O:EtOH (5:1)	6	65
**12**	Fe_3_O_4_@UiO@DAS **5** mg	Reflux	H_2_O:EtOH (5:1)	3	65
**13**	Fe_3_O_4_@UiO@DAS **5** mg	Reflux	H_2_O:EtOH (5:1)	0.5	65
**14**	Fe_3_O_4_@UiO@DAS **10** mg	Reflux	H_2_O:EtOH (5:1)	0.5	83
15	**Fe**_**3**_**O**_**4**_**@UiO@DAS 13 mg**	**Reflux**	**H**_**2**_**O:EtOH (5:1)**	**0.5**	**94**
**16**	Fe_3_O_4_@UiO@DAS 15 mg	Reflux	H_2_O:EtOH (5:1)	0.5	94

Reaction conditions: Benzaldehyde (1a, 1 mmol), malononitrile (2, 1.1 mmol), and 4-hydroxy-6-methyl-2*H*-pyran-2-one (3a, 1 mmol) in the presence of Fe_3_O_4_@UiO@DAS and 2 ml of water–ethanol (5:1) as a green solvent.

Significant values are in [bold].

^a^Isolated yields.

Next, diverse catalysts (4,4′-diamino-2,2′-stilbenedisulfonic acid, MNP@MPS, MNP@MPS@PAA, and Fe_3_O_4_@UiO-66-NH_2_) were used, and the best results were gained using 4,4′-Diamino-2,2′-stilbenedisulfonic acid (73% yield, reflux) (Table 2, entry 5–8).

After that, our synthesized catalyst Fe_3_O_4_@UiO@DAS was used. The best result was gained in the existence of 5 mg of catalyst (68% yield, reflux, 0.5 h) (Table 2, entries 8–13). By studying the amount of catalyst, it was understood that in the presence of 13 mg of the catalyst, the yield of 94% could be gained at 30 min (Table 2, entries 15). By increasing the catalyst amount from 13 to 15 mg, no change in reaction yield was observed (Table 2, entries 16). This result clearly shows that Fe_3_O_4_@UiO@DAS effectively improves the reaction yield. The acidic functional groups (SO_3_H) of 4,4′-diamino-2,2′-stilbenedisulfonic acid as Bronsted acids improve the reaction yield and the nano-particles can also race the reaction up as Lewis acids. However, the optimization results indicate the major active site of the nano-particles to be disulfonic acid functional groups. It should be noted that in all reactions, the catalyst was separated by an external magnet and the final products were filtered out of the mixture.

Additionally, we developed the optimized reaction condition (13 mg of Fe_3_O_4_@UiO@DAS in 3 ml of water–ethanol (5:1) under reflux conditions) for other derivatives of aromatic aldehydes (**1**) and 4-hydroxy-6-methyl-2H-pyran-2-one, 4-hydroxy coumarin and dimedone compound (**3**, **4**, **7**) for the synthesis of the various derivatives of 4*H*-chromenes (**5a–i**, **6a–i**, **8a–h**). The results have been presented in Tables 3, 4 and 5.

**Table 3:** Three-component synthesis of different 2-amino-7-methyl-5-oxo-4-phenyl-4,5-dihydropyrano[4,3-*b*]pyran-3-carbonitrile (5a-i) via condensation of various aldehydes (1), malononitrile (2) and 4-hydroxy-6-methyl-2*H*-pyran-2-one (3) in the presence of Fe_3_O_4_@UiO@DAS.


En	Aldehyde	Compound	Product^a^	Time (min)	Yield^b^ (%)	M.P (°C)
**1**	Benzaldehyde	3	5a	30	94	231–233
**2**	2-Chlorobenzaldehyde	3	5b	20	90	267–269
**3**	4-Chlorobenzaldehyde	3	5c	20	96	227–229
**4**	4-Nitrobenzaldehyde	3	5d	15	95	211–213
**5**	3-Nitrobenzaldehyde	3	5e	15	89	231–232
**6**	Terephthaldehyde	3	5f	40	83	255–257
**7**	4-Methoxybenzaldehyde	3	5g	40	90	213–215
**8**	4-Ethoxybenzaldehyde	3	5h	40	90	202–204
**9**	3-Methylbenzaldehyde	3	5i	40	89	232–234

Reaction conditions: Aldehyde (1, 1 mmol), Malononitrile (2, 1.1 mmol), 4-hydroxy-6-methyl-2*H*-pyran-2-one (3) (1 mmol), and Fe_3_O_4_@UiO@DAS (13 mg) at reflux conditions.

^a^All compounds are known and their structures were established from their melting points compared with authentic samples or literature values.

^b^Isolated yield

**Table 4:** Three-component synthesis of different 2-amino-5-oxo-4-phenyl-4,5-dihydropyrano[3,2-*c*]chromene-3-carbonitrile (6a–i) via condensation of various aldehydes (1), malononitrile (2), and 4-hydroxy coumarin (4) in the presence of Fe_3_O_4_@UiO@DAS.


En	Aldehyde	Compound	Product^a^	Time (min)	Yield^b^ (%)	M.P (°C)
1	Benzaldehyde	4	6a	30	91	256–258
2	4-Chlorobenzaldehyde	4	6b	30	93	255–257
3	2,4-Dichlorobenzaldehyde	4	6c	30	89	261–263
4	4-Nitrobenzaldehyde	4	6d	15	89	254–256
5	3-Nitrobenzaldehyde	4	6e	15	89	256–259
6	4-Methylbenzaldehyde	4	6f	40	95	250–252
7	3-Methylbenzaldehyde	4	6g	40	89	253–255
8	4-Methoxybenzaldehyde	4	6h	40	93	233–235
9	Terephthaldehyde	4	6i	40	85	297–299

Reaction conditions: Aldehyde (1, 1 mmol), Malononitrile (2, 1.1 mmol), 4-hydroxy coumarin (4) (1 mmol), and Fe_3_O_4_@UiO@DAS (13 mg) at reflux conditions.

^a^All compounds are known and their structures were established from their melting points compared with authentic samples or literature values.

^b^Isolated yield.

**Table 5:** Three-component synthesis of different 4*H*-chromene (8a–h) via condensation of various aldehydes (1), malononitrile (2) and dimedone (7) in the presence of Fe_3_O_4_@UiO@DAS.


En	Aldehyde	Compound	Product^a^	Time (min)	Yield^b^ (%)	M.P (°C) Obsd
**1**	Benzaldehyde	7	8a	20	91	235–237
**2**	2-Chlorobenzaldehyde	7	8b	20	93	218–220
**3**	4-Methoxybenzaldehyde	7	8c	25	90	212–214
**4**	4-Methylbenzaldehyde	7	8d	25	90	196–198
**5**	2,4-Dichlorobenzaldehyde	7	8e	20	88	221–223
**6**	Terphthaldehyde	7	8f	30	90	206–208
**7**	3-Nitrobenzaldehyde	7	8g	15	86	210–211
**8**	2-Nitrobenzaldehyde	7	8h	15	86	218–220

Reaction conditions: Aldehyde (1, 1 mmol), Malononitrile (2, 1.1 mmol), dimedone (7, 1 mmol), and Fe_3_O_4_@UiO@DAS (13 mg) at reflux conditions.

^a^All compounds are known and their structures were established from their melting points compared with authentic samples or literature values.

^b^Isolated yield.

Optimized reaction condition also was for the synthesis of 2-amino-7,7-dimethyl-5-oxo-4-aryl-5,6,7,8-tetrahydro-4*H*-chromene-3-carbonitrile, which were collected in Table 4.

It is noteworthy that in all reactions for the synthesis of 4*H*-chromene derivatives (5, 6, 7) syntheses, the reaction of aromatic aldehydes which possessed electron-withdrawing groups are shown to be faster than the reaction of aromatic aldehydes with electron-donating groups. Dimedone required a shorter reaction time compared to the 4-hydroxy-pyrane and 4-hydroxy-coumarin.

For the importance of using heterogeneous catalysts in industrial processes, recyclability of our synthesized catalyst was studied using optimized reaction conditions for synthesis 2-amino-3-cyano-4*H*-chromene via condensation of benzaldehydes (1a), malononitrile (2), and 4-hydroxy-6-methyl-2H-pyran-2-one (3) in the presence of 13 mg of Fe_3_O_4_@UiO@DAS. Our gained results (Table 6) could be used 7 times without a dramatic decrease in its ability. After each reaction, the catalyst was separated by an external magnetic field and washed twice with hot deionized water (10 mL), once with 10 mL ethanol, dried in an oven at 60 °C for 24 h in a vacuum oven reused in the model reaction.

**Table 6:** Recyclability of synthesized Fe_3_O_4_@UiO@DAS for synthesis 2-amino-3-cyano-4H-chromene via condensation of benzaldehydes (1a), malononitrile (2) and 4-hydroxy-6-methyl-2*H*-pyran-2-one (3) in the presence of Fe_3_O_4_@UiO@DAS.

Cycle	1	2	3	4	5	6	7
Yield %	94	94	92	92	89	89	88

## Experimental

### Materials

Our initial materials were provided from Merck and Sigma companies (FeCl_2_.4H_2_O, FeCl_3_.6H_2_O, DMF, NH_4_OH, terephthalic acid, 3-methacryloxypropyltrimethoxy silane (MPS), 4,4′-diaminostilbene-2,2′-disulfonic acid (DAS), Zirconium (IV) chloride, 2,2′-azobisisobutyronitrile (AIBN), and Cyanuric chloride were obtained from Sigma-Aldrich without any purification. The monomer of acrylic acid was supplied by Sigma-Aldrich and was distilled before use.

### Preparation of catalyst

The magnetic nanoparticle (Fe_3_O_4_) was synthesized using the co-precipitation approach^101,102^. After synthesizing it, Fe_3_O_4_@SiO_2_ (1 g) was dispersed in dry EtOH and NH_4_OH (2 mL) was added to the mixture. Then, MPS (10 mL) was gradually added to the above mixture at 60 °C, and for 48 h the mixture was stirred. Using an external magnet, magnetic nanoparticles were collected, washed, and dried for 24 h under vacuum conditions.

Next, 0.4 of synthesized magnetic nanoparticles (which we called MNP@MPS) was dispersed in MeOH (30 mL), and acrylic acid (0.4 g) was added to it. After purging Ar into the mixture (for 20 min), AIBN (0.1) was added to it and the mixture was stirred for 24 h at 70 °C. The final product (MNP@PAA) was collected by an external magnet, washed, and dried under vacuum conditions.

To synthesize of Fe_3_O_4_@UiO-66, synthesized MNP@MPS@PAA (0.2 g ) was added to DMF (30 mL) and sonicated for 30 min. Next, by adding 0.53 g of zirconium (IV) chloride (0.53 g) and terephthalic acid (0.38 g) to the mixture, it was left to stir for 2 h. after 2 h, the autoclave was used to heat the mixture (120 °C, 1 day). Resulted product was centrifuged and washed. Also, chloroform was used for the exchanging of the solvent. Finally, Magnetic UiO-66-NH_2_ was heated to 120 °C and kept under vacuum condition for one week.

To access the final catalyst Fe_3_O_4_@UiO@DAS, Fe_3_O_4_@UiO-66-NH_2_ (1.0 g) was dispersed in 20 mL of dry THF in a round bottom flask and sonicated for 20 min. Then, TCT (2.0 g, 10 mmol) was added to the mixture. Afterward, 4,4′-diamino-2,2′-stilbenedisulfonic acid (2.9 g, 14 mmol) was added gradually to the mixture under stirring at 0 °C. Then, K_2_CO_3_ (2.0 g, 14 mmol) was added in the next step to the mixture and stirred for 3–4 h at room temperature and then was refluxed at 50 °C for 24 h. The prepared Fe_3_O_4_@UiO-66-NH_2_ was magnetically separated and washed three times with methanol and chloroform to remove any excess reagents and then dried at 60 °C for 24 h in a vacuum oven.

### Synthesis of 4H‑chromene derivatives

A glass vial was successively charged with different enolizable compounds (1 mmol), aldehydes (1 mmol), and active methylene nitrile (1.1 mmol) in the presence of Fe_3_O_4_@UiO@DAS (13 mg), in water–ethanol (5:1, 3 mL) at reflux temperature. The reaction mixture was stirred for the appropriate time brought in Tables 3, 4, and 5. After reaction completion, which was controlled by Thin Layer Chromatography (TLC) test (using EtOAc/n-Hexane, 1:3 as solvent), the catalyst was separated by a magnet, and the obtained solid product was filtered. In the case of impurities, the obtained product was recrystallized from ethanol.

## Conclusions

Zr-cluster-based MOFs have fascinating characteristics and have huge variety of applications. To increase their applications, hybrid nanomaterials based on MOFs have synthesized. Synthesizing hybrid materials make it possible to use the advantages of both parts in their structures. In this project, to use the advantages of MOFs (such as high chemical stability) and magnetic nanoparticles (such as simple separation process) we have decided to synthesize a novel magnetic UiO-66 functionalized with 4,4′-diamino-2,2′-stilbenedisulfonic. This modified MOF characterized by various techniques, including FT-IR, XRD, BET, TGA, TEM, EDX, and SEM. To investigate the applications of our modified magnetic MOF, it was used for the synthesis of 4*H*-chromene skeletons via a one-pot three components reaction in a green solvent. This non-hazardous, recyclable, effective, and appropriate catalyst allowed quick and effective access to diverse 4*H*-chromene derivatives. The synthesized catalyst can be extracted from the reaction media by an external magnetic field and recycled. Briefly, the absence of harsh conditions in the synthesis of catalyst, reusability, mild reaction conditions, and up to 96% yields of products are advantageous of our introduced method.
